# Anatomical Atlas of Kinase Responsiveness to Weight Gain: Adipose Depot Reprogramming in Diet-Induced Adiposity

**DOI:** 10.3390/metabo16050318

**Published:** 2026-05-09

**Authors:** Wang-Hsin Lee, Zachary A. Kipp, Sally N. Pauss, Genesee J. Martinez, Mei Xu, Terry D. Hinds

**Affiliations:** 1 Drug & Disease Discovery D3 Research Center, Department of Pharmacology and Nutritional Sciences, College of Medicine, University of Kentucky, 760 Press Ave, Healthy Kentucky Research Building (HKRB) 224, Lexington, KY 40508, USA; wang-hsin.lee@uky.edu (W.-H.L.); zachary.kipp@uky.edu (Z.A.K.); sally.pauss@uky.edu (S.N.P.); genesee.martinez@uky.edu (G.J.M.); 2 Barnstable Brown Diabetes Center, University of Kentucky College of Medicine, Lexington, KY 40508, USA; 3 Center for Clinical and Translational Science, University of Kentucky College of Medicine, Lexington, KY 40508, USA; mxu222@uky.edu; 4 Markey Cancer Center, University of Kentucky, Lexington, KY 40508, USA

**Keywords:** kinome, PamGene, obesity, MASLD, CKM syndrome, inflammation, visceral adipose tissue, subcutaneous adipose tissue, white fat, brown fat

## Abstract

**Highlights:**

**What are the main findings?**

- We observed that the location of adipose tissue depots in the body is directly linked to how their kinases respond to adiposity.
- We identified kinase markers unique to adipose tissue depots linked to adiposity.

**What are the implications of the main findings?**

- A more profound and comprehensive understanding of the regulatory functions of kinases in adipose tissues across various anatomical locations.
- A comprehensive understanding of kinase pathways involved in adipose tissue expansion could facilitate the development of future therapeutic interventions for obesity and metabolic disorders.

**Abstract:**

Background/Objectives: Adipose tissue depots located at different anatomical sites exert differential functions in response to adiposity and glucose intolerance. These fat depots exhibit distinct metabolic signaling patterns that may influence pathological fat accumulation, thereby affecting the efficacy of anti-obesity interventions. Nonetheless, the mechanisms underpinning depot-specific signaling and pathway responsiveness remain insufficiently understood. Methods: Kinase activity was characterized during the progression of adiposity across five adipose tissue depots in obese versus lean mice using the advanced PamGene kinome technology. Furthermore, kinase pathways in human preadipocytes and mouse 3T3-L1 preadipocytes were analyzed and compared with those in their differentiated, mature adipocytes. The kinases most significantly altered across adipose tissue depots were identified, revealing depot-specific combinations of hyperactive and hypoactive kinase pathways involved in adiposity. Results: Our findings demonstrate distinct kinase families that regulate specific fat depots, with potential implications for drug discovery and therapeutic resistance. Conclusions: This research presents a comprehensive adipokinome atlas, elucidates potential targets for developing fat-depot-specific anti-obesity therapies, and offers novel insights into the functional heterogeneity of adipose tissues.

## 1. Introduction

The increasing prevalence of obesity persists despite the availability of contemporary therapeutic interventions [1]. Obesity is defined as the excessive accumulation of adipose tissue and is intricately linked to metabolic disturbances, including insulin resistance, diabetes, and MASLD (Metabolic Dysfunction-Associated Steatotic Liver Disease) [2,3,4]. Notably, a subset of individuals with obesity maintains metabolic health throughout their lives [5], though more studies are needed to better understand the underlying mechanisms. It has been reported that individuals with obesity exhibit considerable variability in fat distribution, leading to divergent metabolic outcomes and disease risk profiles [6], thereby indicating that the anatomical distribution of adipose tissue plays a crucial role in determining metabolic consequences.

To investigate the differences in white adipose tissue (WAT), the heterogeneity between visceral adipose tissue (VAT) and subcutaneous adipose tissue (SCAT) has been thoroughly examined. Anatomically, VAT consists of fat depots located within the abdominal cavity, including adipose tissue surrounding the reproductive organs, liver, pancreas, kidneys, and intestines. Due to its proximity to the liver and intestines, most VAT receives its blood supply from the portal vein, facilitating direct metabolic communication with the liver [7]. Conversely, SCAT comprises fat depots stored directly beneath the skin and is typically found in the limbs and abdominal wall. SCAT is primarily supplied by the peripheral systemic circulation, resulting in limited direct interactions with major metabolic organs such as the liver. Compared to VAT, SCAT demonstrates lower vascular density, reduced blood supply, and lower tissue temperatures. From a physiological standpoint, VAT exhibits higher lipolytic activity and is more strongly associated with impaired insulin sensitivity than SCAT, as reported by [8]. VAT also exhibits increased adipocyte-macrophage interactions and contributes significantly to pro-inflammatory responses [8,9]. Adipokines and free fatty acids released from VAT can enter the portal circulation and directly influence hepatic metabolism, which may elucidate VAT’s role in metabolic and inflammatory regulation [7]. Furthermore, adipocytes in VAT tend to be larger than those in SCAT, a characteristic associated with reduced insulin sensitivity and increased cardiovascular risk [7]. Zhang et al. created an adipose tissue atlas to identify human-like brown adipose tissue (BAT) and WAT with beige depots in mice [10]. This work demonstrated several gene markers for the various fat depots.

In this study, we used kinase signaling mechanisms to establish a kinase pathway atlas of adipose tissue responsiveness to adiposity. Kinase activity has become a major focus of modern research, as kinases are key regulators of biological and physiological processes. Receptor protein tyrosine kinases (PTK), for example, can initiate signaling cascades through phosphorylating downstream target proteins [11]. In contrast, serine-threonine kinases (STKs) often function as intermediates or downstream signal carriers, becoming activated by phosphorylation and subsequently phosphorylating intracellular substrates [12]. A well-characterized example is the insulin receptor and its signaling pathway [13,14]. The insulin receptor is a PTK that initiates signaling by phosphorylating insulin receptor substrate (IRS) and a cascade of other substrates [2,14]. This activation propagates the signaling cascade, leading to increased phosphorylation of STKs, including protein kinase B (AKT), mechanistic target of rapamycin (mTOR), and extracellular signal-regulated kinase (ERK) [14]. A kinase activity analysis of PTKs and STKs can quickly identify activation of known and even unknown signaling pathways, which has proven essential for understanding complex signaling networks. However, traditional approaches, such as immunoblotting probing for phosphoproteins, are time-consuming and cannot show real-time kinase activity. Recently, a state-of-the-art technology, PamGene PamStation, has emerged, enabling efficient, real-time analysis of kinase activity for hundreds of kinases simultaneously [15,16].

By incorporating PamGene PamStation technology, our study developed a comprehensive framework for understanding how kinase signaling in adipose tissue depots responds to perturbations in metabolic health. Nonetheless, most research has focused on broad comparisons between these two categories, even though each includes multiple anatomically and functionally distinct depots. For instance, VAT comprises epididymal (eWAT), retroperitoneal (rWAT), and mesenteric (mWAT) adipose tissues. Considering these depots as a singular category may obscure depot-specific mechanisms and hinder the development of targeted therapeutic strategies. Accordingly, this study aimed to investigate signaling responsiveness and pathway regulation not only at the VAT and SCAT levels but also within individual adipose depots by profiling adiposity-induced alterations in kinase activity. This work and methodology offer valuable insights into depot-specific pathway modifications and identify potential therapeutic targets for the selective modulation of fat accumulation within specific adipose depots.

## 2. Materials and Methods

Animals and Tissue Collection

Eight-week-old male C57BL/6 mice (Jackson Lab, Bar Harbor, ME, USA) were randomly assigned to groups fed a normal chow diet (NCD) (n = 6) or a 60% high-fat diet (HFD) (Research Diets, Cat# D12492) (n = 7) for 20 weeks. EchoMRI was performed at the end of the study to measure fat and lean body mass. Mice were fasted overnight before tissue collection, and tissue samples, including adipose tissue, were collected and weighed. Cages were distributed across the rack to avoid location bias, and all procedures were performed at consistent times of day to minimize circadian variation. No mice were excluded from the subsequent analysis. Exclusion criteria were predefined based on humane endpoints, including reduced activity or other clinical signs of distress observed during daily monitoring. Each mouse was considered the experimental unit for this study. Animals were allocated to experimental groups by an independent investigator. Given the nature of the experimental design, investigators administering the diet were aware of the group allocations. The outcomes of all experiments measured are described in detail below. The animal study was approved by the Institutional Animal Care and Use Committee of the University of Kentucky (protocol code 2020-3665).

Mouse Adipocytes Differentiation

Undifferentiated and differentiated 3T3-L1 mouse fibroblasts were used to represent the kinome responsiveness in mouse adipocytes during adiposity. The cells were cultured in full media, which contains DMEM (1X) + GlutaMAX-1 media (Gibco, Waltham, MA, USA Cat# 10566-016) supplemented with 10% bovine calf serum (Cytiva, Marlborough, MA, USA, Cat# SH30073.03) and 1% Gibco™Antibiotic-Antimycotic (Gibco, Cat# 15240-062), and the incubator setting was 37 °C in a 5% CO2. To differentiate 3T3-L1, the cells were cultured in full media to 100% confluency, followed by the change of full media to MDI differentiation induction media containing DMEM (1X) + GlutaMAX-1 media (Gibco, Cat# 10566-016) supplemented with 10% FBS (Alkali Scientific Inc., Fort Lauderdale, FL, USA, Cat# FB-72), 1% Gibco™Antibiotic-Antimycotic, 500 μM IBMX, 1 μM Dexamethasone, and 167 µM insulin for 2 days. Then, the MDI media was replaced with insulin differentiation media containing DMEM (1X) + GlutaMAX-1 (Gibco, Cat# 10566-016), supplemented with 10% FBS (Alkali Scientific Inc., Cat# FB-72), 1% Gibco™ Antibiotic-Antimycotic, and 167 µM insulin for 4 days. The insulin differentiation media were replaced with DMEM (1X) + GlutaMAX-1 (Gibco, Cat# 10566-016), supplemented with 10% FBS (Alkali Scientific Inc., Cat# FB-72) and 1% Gibco™ Antibiotic-Antimycotic, for 1 day; then the cells were harvested for protein extraction.

Human Primary Visceral Preadipocytes Differentiation

HPA-v preadipocytes (ScienCell, Carlsbad, CA, USA, Cat# 7210) were cultured in preadipocyte media (ScienCell, Cat# 7211) with Preadipocyte Growth Supplement (ScienCell, Cat #7252) for regular growing. Before differentiation began, the undifferentiated cells were harvested as the control group. The differentiation of HPA-v began by switching to differentiation media (ScienCell, Cat# 7221) supplemented with an additive kit (ScienCell, Cat #7232), and the cells were incubated in this medium for 14 days. Differentiation media were refreshed every 2 days.

Cell Sample Extraction

The protein was extracted with Mammalian Extraction Reagent (M-PER) (Thermo Fischer Scientific, Waltham, MA, USA, CAT #78503) supplemented with Halt Phosphatase Inhibitor (Thermo Fischer Scientific, CAT #78428) and Protease Inhibitor Cocktail (Sigma-Aldrich, St. Louis, MO, USA, CAT #P2714). Cell lysates were homogenized using a pellet pestle and then centrifuged. The supernatant containing the protein lysate was collected. Protein concentrations were determined in triplicate by using the Pierce BCA Protein assay kit (Thermo Fischer Scientific, CAT #23225).

PamGene PamStation Assays

Kinase activity was measured in the samples using the PamGene PamStation 12 instrument, as previously described [13,15,17,18,19,20,21]. Samples were pooled within their treatment group for mouse adipose tissues and processed individually for mouse adipocytes and human primary visceral adipocytes. Protein samples from the adipose tissues were prepared using Qiagen TissueLyser and M-PER protein extraction reagent (Thermo Fisher Scientific, 78501) supplemented with protease inhibitor (Sigma-Aldrich, P2714) and phosphatase inhibitor (Thermo Fisher Scientific, 78420). The samples were centrifuged to collect the supernatant. Protein concentrations were measured with Pierce Microplate BCA Protein Assay Kit (Thermo Fisher Scientific, 23252) in triplicate. Per array for serine-threonine kinase (STK) assay (PamGene, ’s-Hertogenbosch, the Netherlands, 32501), 1 microgram of protein was added, and 5 micrograms of protein was added per array for protein-tyrosine kinase (PTK) assay (PamGene, 32516). 4 mM adenosine triphosphate (ATP) and fluorescently labeled antibodies (PamGene, 32112.5 PTK, 32201.3 STK) were added with the protein samples for the detection of phosphorylation conditions of the peptide substrates. Mouse adipose tissue samples were run in technical triplicate using 3 PamChips for both PTK and STK assays, while biological triplicate was used for cell samples. Evolve 3 (PamGene, Version: 3.1.0.5) software uses a charge-coupled device (CCD) camera and light-emitting diode (LED) imaging system to measure phosphorylation levels of each peptide substrate. Signal intensities were recorded every 5 min over a 60 min period, using exposure times of 10, 20, 50, and 100 milliseconds. In total, 94 cycles were performed for PTK and 124 cycles for STK assays. Raw images were exported for data analysis. Kinase mapping procedures are described in the BioNavigator Bioinformatics Analysis and Kinome Random Sampling Analysis (K.R.S.A) sections.

PamGene PamStation Kinome Data Analysis

Array images from the runs were analyzed with Tercen BioNavigator (PamGene). The across-chip coefficient of variation (CV) of high signals for technical replicates indicates low variability across chips. The fold change (FC) for each phospho-substrate peptide was computed using signal ratios and averaged across three PamChips. Threshold criteria, based on previous literature [17,18,19,20,21], require phosphorylation signals to be ≥30% (FC ≥ 1.30 or FC ≤ 0.70) to qualify as differential phosphorylation. Linear regression slopes were used to generate time-series phosphorylation intensities for comparison between groups (e.g., experimental vs. control). Data exported from BioNavigator after signal intensity calculations were used for further analysis. Upstream kinase identification was conducted with BioNavigator Upstream Kinase Analysis (UKA) (PamGene), which predicts the kinases most likely responsible for phosphorylation events on the PamChip over time. BioNavigator employs a mapping file with experimentally confirmed and predicted substrates for each kinase, ranked from 0 (high confidence, based on in vitro or in vivo experiments) to 12 (low confidence, based on predictive models). The plots for Measurements Extensively Of Winner (MEOW) were generated with Prism (GraphPad) as previously described [15,18,20]. In brief, they were calculated [Log2 Fold Change (FC) of kinase substrates * (multiplied by) Δ confidence (experimental hits/mean hits of 2000 random sampling iterations)].

Kinome Random Sampling Analysis (K.R.S.A.)

The signal intensity and saturation data were exported to an Excel file for further analysis using the Kinome Random Sampling Analysis (K.R.S.A.) package in RStudio (Version: 2026.01.1+403) [22]. Phosphopeptides that were undetectable or exhibited nonlinear behavior (R2 < 0.80) were excluded from subsequent analyses.

CORAL Kinome Phyla Trees

The kinome phyla tree was constructed using CORAL [23]. The tree values were generated and exported from BioNavigator. Each node corresponds to a kinase, grouped by family. Only kinases measured in our analysis are included. The size of each node indicates the mean final score for statistical significance, while the color reflects the median kinase statistic, showing kinase activity. The ranges for node size and color are determined for each tissue and cell type.

Statistics

Kinase activity graphs from PamGene show individual points for each measured kinase target substrate. All raw data from the PamStation were processed and analyzed using Tercen BioNavigator, which provided the data for individual kinases. Kinase data from the PamStation were also analyzed using the kinase random sampling analysis (KRSA) package in R, which is compatible with both individual kinases and kinase families. Results from both pipelines were used and presented. A Unpaired *t*-test was used to compare the animal and tissue weights across groups (Table 1). *p*-values < 0.05 were considered statistically significant.

## 3. Results

The mice treated with HFD showed significantly increased body weight (BW) and tissue weights in total fat mass, iWAT, eWAT, mWAT, rWAT, BAT, liver, heart, pancreas, spleen, and kidney (Table 1, upper table). Blood glucose levels were significantly increased in the HFD-treated group (Table 1, upper table). However, after each mouse tissue weight was normalized by body weight per mouse, the statistical significance for liver and spleen weights was no longer present (Table 1, bottom table).

**Figure 1:**
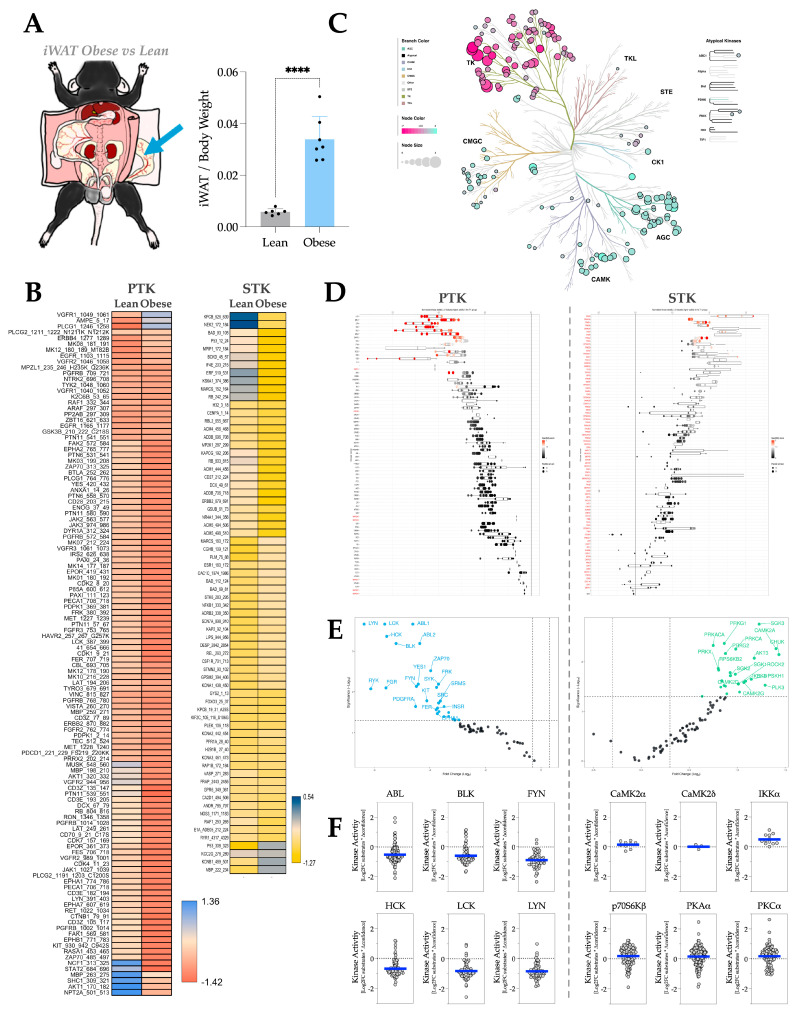
iWAT kinase activity profile in response to HFD-induced adiposity. (**A**) Diagram showing the anatomical location of iWAT in a C57BL/6 mouse body and the fat pad weight change of iWAT by body weight in response to HFD treatment. [****, *p* < 0.0001; n = 6 for NCD and n = 7 for HFD; unpaired *T*-test; Mean ± S.D.] (**B**) Heatmaps showing average differentially phosphorylated PTK (**left**) and STK (**right**) substrates (averaged across three technical replicates). (**C**) Paralogous phylogenetic correlations between differentially activated kinases in response to HFD treatment. The iWAT PTK and STK data from mice treated with NCD or HFD were compared and analyzed. Node color represents the median kinase statistic (kinase activity), and node size represents the median final score (significance) on the bubble plot of the paralogous phylogenetic trees. (**D**) The individual PTK (**left**) and STK (**right**) activities, plotted using normalized kinase statistics, show the trend in kinase activity changes in response to HFD treatment. (**E**) Volcano plots showing the Log_2_ fold changes and significance of individual PTK (**left**) or STK (**right**). Green-colored kinases exhibit hyperactivity that passes both the significance and Log_2_FC cutoffs, whereas blue-colored kinases show hypoactivity that also meets these criteria. (**F**) MEOW plots showing representative top-changed PTK (**left**) or STK (**right**) activities. The blue line in MEOW plots indicates the overall activity among all the included substrates.

To explore kinase activity, we performed kinome analysis using PamGene PamStation. In mouse iWAT, a SCAT (Figure 1A), HFD-induced adiposity reduces most of the phosphorylation on PTK substrates and a dichotomous regulation of STK substrates on the PamChips, shown in the heatmaps (Figure 1B). To show the individual kinase activities, we visualized the data with a phylogenetic tree and waterfall plots. The phyla tree diagram presents the paralogous phylogenetic correlations between differentially activated kinases in clusters (Figure 1C), where we found that the activity of PTK (shown as the cluster named TK in Figure 1C) decreased, and the activity of STK (shown as clusters other than TK in Figure 1C) increased in response to adiposity. The waterfall plots show trends in activity changes for individual PTKs (Figure 1D, left) or STKs (Figure 1D, right) in response to adiposity, and the heatmap results are comparable to those visualized in the phyla tree (Figure 1C). Shifts in predicted kinase activity, as shown phylogenetically in Panel C, are also depicted individually in Panel 1D. To identify the most changed kinases, we visualized the data using volcano plots, in which the most significant kinases were highlighted in color with their names (Figure 1E). After identifying the most changed kinases, we selected 6 representative most-changed PTKs and 6 representative most-changed STKs and created the MEOW plots using the phosphorylation levels of their substrates (Figure 1F). The MEOW plots identified that PTKs like Abelson murine leukemia viral oncogene homolog 1 (ABL), B lymphocyte kinase (BLK), Proto-oncogene Syn (FYN), Hematopoietic cell kinase (HCK), Leukocyte C-terminal Src kinase (LCK), and Lck/Yes-related novel protein tyrosine kinase (LYN) are significantly hypoactive in iWAT in adiposity. Whereas STKs like CaM kinase II subunit alpha (CaMK2α), CaM kinase II subunit delta (CaMK2δ), I-kappa-B kinase alpha (IKKα), S6K-beta-2 (p70S6Kβ), cAMP-dependent protein kinase catalytic subunit alpha (PKAα), and Protein kinase C alpha (PKCα) are hyperactive in the iWAT in adiposity. The data were visualized for the remaining adipose depots and adipocyte cell lines with the same method and arrangement.

**Figure 2:**
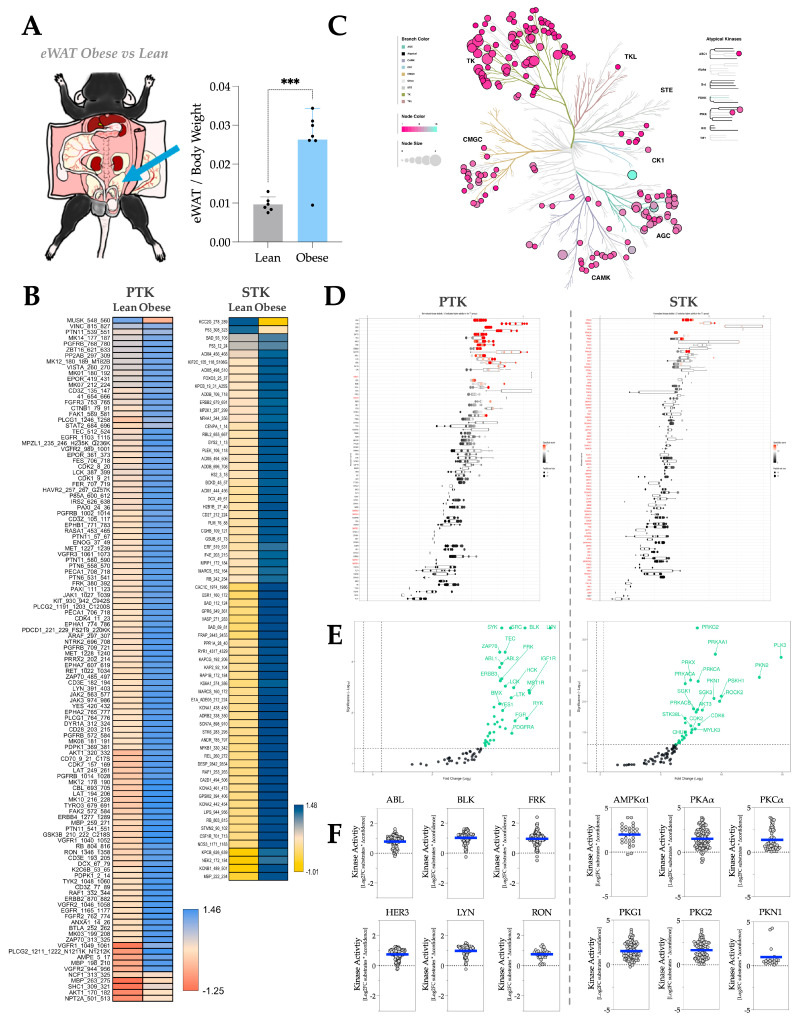
eWAT kinase activity profile in response to HFD-induced adiposity. (**A**) Diagram showing the anatomical location of eWAT in a C57BL/6 mouse body and the fat pad weight change of eWAT by body weight in response to HFD treatment. [Statistical analysis, unpaired *T*-test; ***, *p* < 0.001; n = 6 for NCD and n = 7 for HFD; Mean ± S.D.] (**B**) Heatmaps showing average differentially phosphorylated PTK (**left**) and STK (**right**) substrates (averaged across three technical replicates). (**C**) Paralogous phylogenetic correlations between differentially activated kinases in response to HFD treatment. The eWAT PTK and STK data from mice fed an NCD or HFD were compared and analyzed. Node color represents the median kinase statistic (kinase activity), and node size represents the median final score (significance) on the bubble plot of the paralogous phylogenetic trees. (**D**) The individual PTK (**left**) and STK (**right**) activities, plotted using normalized kinase statistics, show the trend in kinase activity changes in response to HFD treatment. (**E**) Volcano plots showing the Log2 fold changes and significance of individual PTK (**left**) or STK (**right**). Green-colored kinases exhibit hyperactivity that passes both the significance and Log2FC cutoffs. (**F**) MEOW plots showing representative top-changed PTK (**left**) or STK (**right**) activities. The blue line in MEOW plots indicates the overall activity among all the included substrates.

In mouse eWAT, a well-known VAT (Figure 2A), HFD-induced adiposity greatly increases phosphorylation of PTK and STK substrates, as shown in the heatmaps (Figure 2B). The phylogenetic tree shows that the activities of both PTK and STK increase significantly with adiposity, with a more pronounced effect in STK (Figure 2C). The waterfall and volcano plots show the same trend in individual PTK and STK activities as those shown in the phyla tree (Figure 2D,E). With MEOW plots (Figure 2F), we show the most hyperactive PTKs in adiposity, including ABL, BLK, FYN-related kinase (FRK), Receptor tyrosine-protein kinase erbB-3 (HER3), Lck/Yes-related novel protein tyrosine kinase (LYN), and Macrophage-stimulating protein receptor (RON). Whereas the most-hyperactive STKs in adiposity include 5′-AMP-activated protein kinase catalytic subunit alpha-1 (AMPKα1), PKAα, PKCα, cGMP-dependent protein kinase 1 (PKG1), cGMP-dependent protein kinase 2 (PKG2), and Serine/threonine-protein kinase N1 (PKN1).

**Figure 3:**
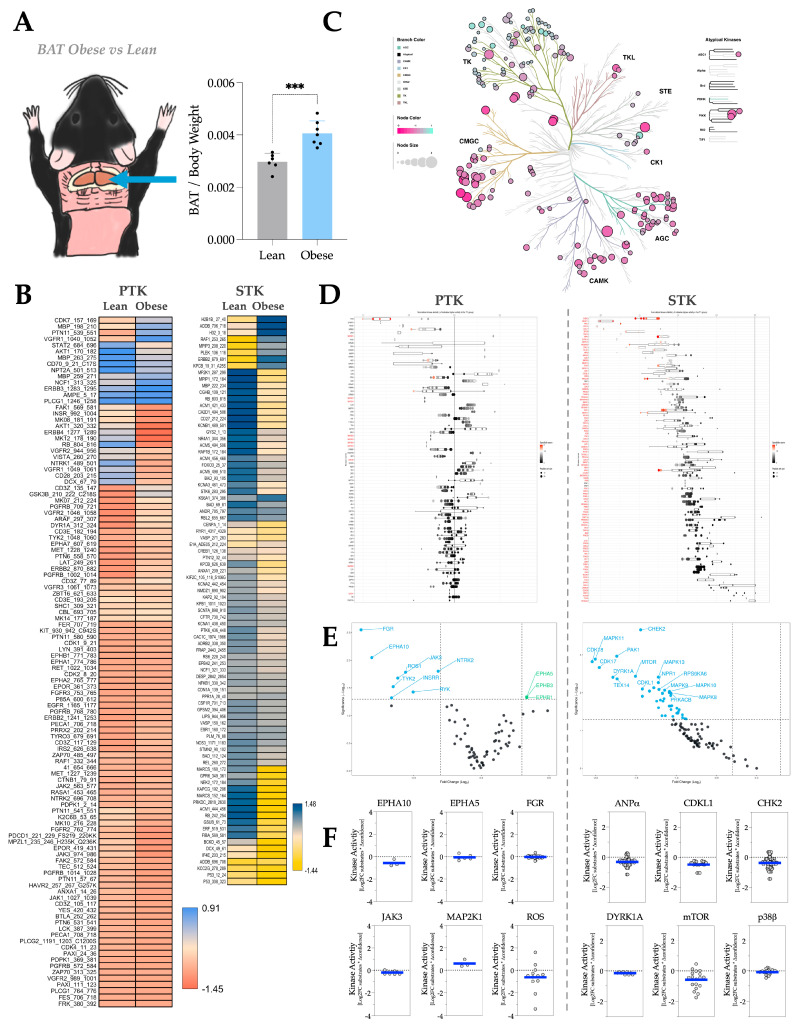
BAT kinase activity profile in response to HFD-induced adiposity. (**A**) Diagram showing the anatomical location of BAT in a C57BL/6 mouse body and the fat pad weight change of BAT by body weight in response to HFD treatment. [Statistical analysis, unpaired *T*-test; ***, *p* < 0.001; n = 6 for NCD and n = 7 for HFD; Mean ± S.D.] (**B**) Heatmaps showing average differentially phosphorylated PTK (**left**) and STK (**right**) substrates (averaged across three technical replicates). (**C**) Paralogous phylogenetic correlations between differentially activated kinases in response to HFD treatment. The BAT PTK and STK data from mice treated with NCD or HFD were compared and analyzed. Node color represents the median kinase statistic (kinase activity), and node size represents the median final score (significance) on the bubble plot of the paralogous phylogenetic trees. (**D**) The individual PTK (**left**) and STK (**right**) activities, plotted using normalized kinase statistics, show the trend in kinase activity changes in response to HFD treatment. (**E**) Volcano plots showing the Log2 fold changes and significance of individual PTK (**left**) or STK (**right**). Green-colored kinases exhibit hyperactivity that passes both the significance and Log2FC cutoffs, whereas blue-colored kinases show hypoactivity that also meets these criteria. (**F**) MEOW plots showing representative top-changed PTK (**left**) or STK (**right**) activities. The blue line in MEOW plots indicates the overall activity among all the included substrates.

In mouse brown adipose tissue (BAT) (Figure 3A), PTK substrates appear to be differentially phosphorylated, and STK substrates are overall hypo-phosphorylated in response to adiposity (Figure 3B). The phyla tree shows that some PTK activities increase, whereas others decrease, with adiposity (Figure 3C). In contrast, STKs are significantly decreased in response to adiposity (Figure 3C). The waterfall and volcano plots show the same trend in individual PTK and STK activities, as presented in the phyla tree (Figure 3D,E). We then generated MEOW plots (Figure 3F), where we identified that the most-changed PTKs in adiposity include Ephrin type-A receptor 10 (EPHA10), Ephrin type-A receptor 5 (EPHA5), Proto-oncogene c-Fgr (FGR), Janus kinase 3 (JAK3), Dual specificity mitogen-activated protein kinase kinase 1 (MAP2K1), and Receptor tyrosine kinase c-ros oncogene 1 (ROS). Whereas the most-changed STKs in adiposity include Atrial natriuretic peptide receptor type A (ANPα), Cyclin-dependent kinase-like 1 (CDKL1), Checkpoint kinase 2 (CHK2), Dual specificity YAK1-related kinase (DYRK1A), Mammalian target of rapamycin (mTOR), and Mitogen-activated protein kinase 11 (p38β).

**Figure 4:**
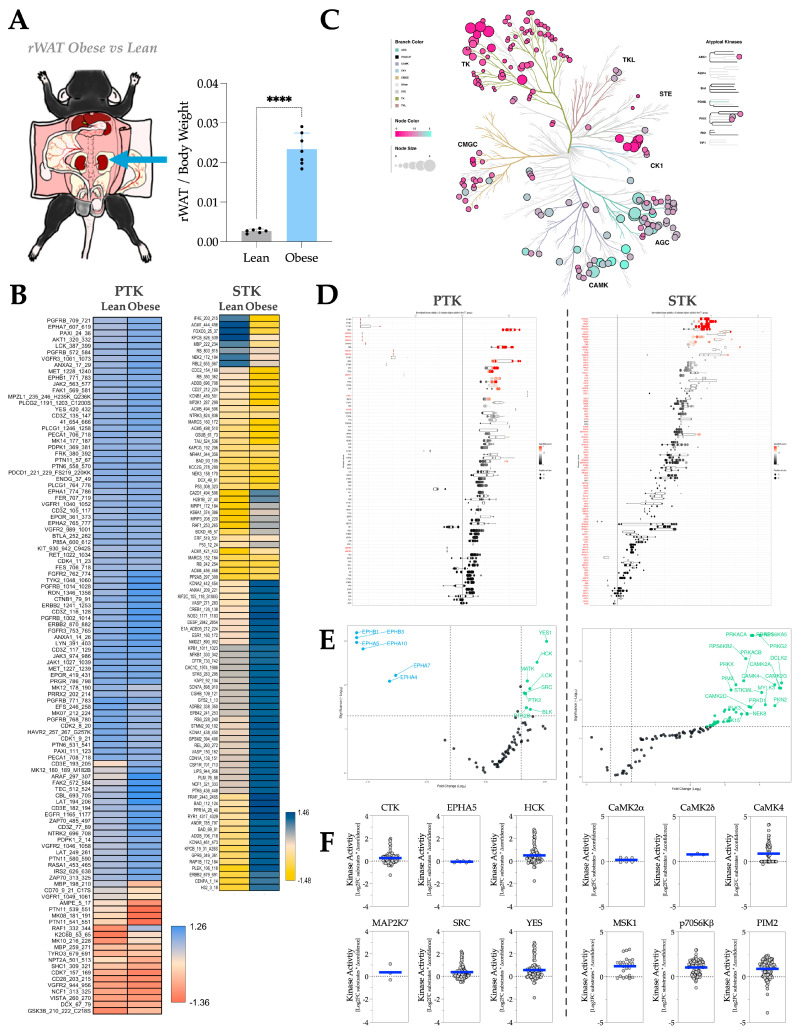
rWAT kinase activity profile in response to HFD-induced adiposity. (**A**) Diagram showing the anatomical location of rWAT in a C57BL/6 mouse body and the fat pad weight change of rWAT by body weight in response to HFD treatment. [Statistical analysis, unpaired *T*-test; ****, *p* < 0.0001; n = 6 for NCD and n = 7 for HFD; Mean ± S.D.] (**B**) Heatmaps showing average differentially phosphorylated PTK (**left**) and STK (**right**) substrates (averaged across three technical replicates). (**C**) Paralogous phylogenetic correlations between differentially activated kinases in response to HFD treatment. The rWAT PTK and STK data from mice treated with NCD or HFD were compared and analyzed. Node color represents the median kinase statistic (kinase activity), and node size represents the median final score (significance) on the bubble plot of the paralogous phylogenetic trees. (**D**) The individual PTK (**left**) and STK (**right**) activities, plotted using normalized kinase statistics, show the trend in kinase activity changes in response to HFD treatment. (**E**) Volcano plots showing the Log2 fold changes and significance of individual PTK (**left**) or STK (**right**). Green-colored kinases exhibit hyperactivity that passes both the significance and Log2FC cutoffs, whereas blue-colored kinases show hypoactivity that also meets these criteria. (**F**) MEOW plots showing representative top-changed PTK (**left**) or STK (**right**) activities. The blue line in MEOW plots indicates the overall activity among all the included substrates.

In mouse rWAT, the VAT surrounding the kidney (Figure 4A), HFD-induced adiposity has a dichotomous effect on PTK substrate phosphorylation, predominantly increasing the phosphorylation of STK substrates (Figure 4B). The phyla tree shows that the overall activities of both PTK and STK increase significantly with adiposity, with STK exhibiting a stronger increase than PTK (Figure 4C). The waterfall and volcano plots reiterate these findings, highlighting individual kinases that are significantly altered (Figure 4D,E). Notably, although the overall PTK activity is increased, a small group of Ephrin type-A receptor (EPHA) kinases is significantly decreased in activities in adiposity (Figure 4D, left and Figure 4E, left). Using MEOW plots analysis (Figure 4F), we identify the most significantly changed PTKs in adiposity as CSK homologous kinase (CTK), EPHA5, HCK, Dual specificity mitogen-activated protein kinase kinase 7 (MAP2K7), SRC (proto-oncogene tyrosine-protein kinase SRC), and YES (YES Proto-Oncogene 1, SRC Family Tyrosine Kinase). Whereas the most-hyperactive STKs in adiposity include CaMK2α, CaMK2δ, Calcium/calmodulin-dependent protein kinase type IV (CaMK4), S6K-alpha-5 (MSK1), p70S6Kβ, and Serine/threonine-protein kinase pim-2 (PIM2).

**Figure 5:**
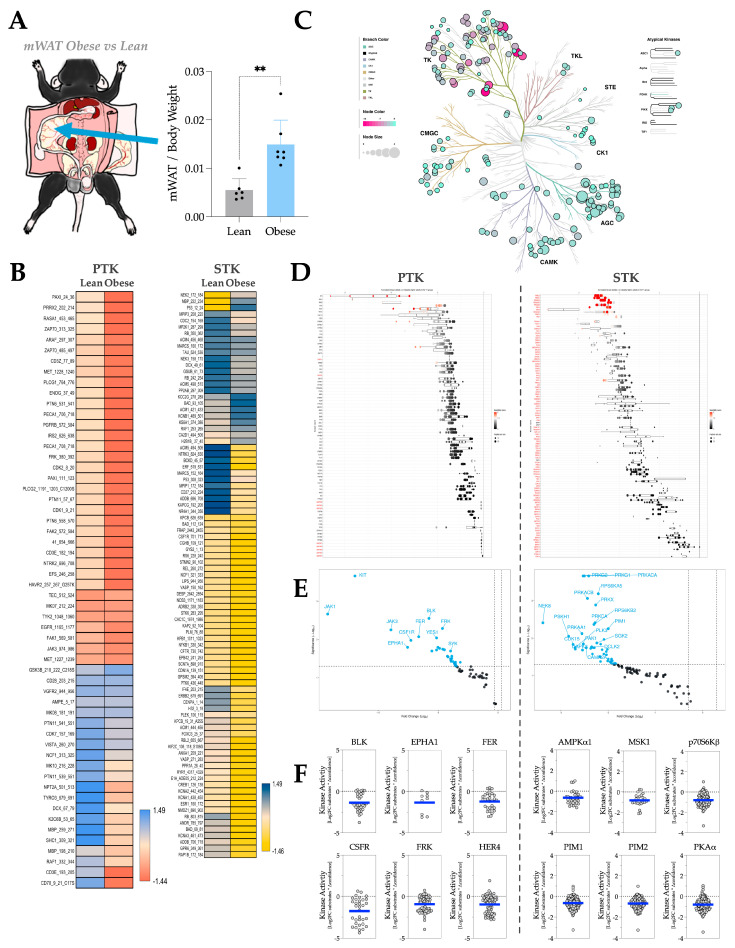
mWAT kinase activity profile in response to HFD-induced adiposity. (**A**) Diagram showing the anatomical location of mWAT in a C57BL/6 mouse body and the fat pad weight change of mWAT by body weight in response to HFD treatment. [Statistical analysis, unpaired *T*-test; **, *p* < 0.01; n = 6 for NCD and n = 7 for HFD; Mean ± S.D.] (**B**) Heatmaps showing average differentially phosphorylated PTK (**left**) and STK (**right**) substrates (averaged across three technical replicates). (**C**) Paralogous phylogenetic correlations between differentially activated kinases in response to HFD treatment. The mWAT PTK and STK data from mice treated with NCD or HFD were compared and analyzed. Node color represents the median kinase statistic (kinase activity), and node size represents the median final score (significance) on the bubble plot of the paralogous phylogenetic trees. (**D**) The individual PTK (**left**) and STK (**right**) activities, plotted using normalized kinase statistics, show the trend in kinase activity changes in response to HFD treatment. (**E**) Volcano plots showing the Log2 fold changes and significance of individual PTK (**left**) or STK (**right**). Blue-colored kinases exhibit hypoactivity that passes both the significance and Log2FC cutoffs. (**F**) MEOW plots showing representative top-changed PTK (**left**) or STK (**right**) activities. The blue line in MEOW plots indicates the overall activity among all the included substrates.

In mouse mWAT, the VAT attached to the intestine (Figure 5A), HFD-induced adiposity decreases the phosphorylation of most PTK and STK substrates (Figure 5B). The phyla tree shows that the overall activities of both PTK and STK significantly decrease in response to adiposity (Figure 5C). The waterfall and volcano plots show changes in activity for individual kinases within the PTK and STK families, demonstrating these decreases at the individual level (Figure 5D,E). With MEOW plots (Figure 5F), we identify that the individual PTKs in adiposity include BLK, Ephrin type-A receptor 1 (EPHA1), Feline encephalitis virus-related kinase FER (FER), Macrophage colony-stimulating factor 1 receptor (CSFR), FYN-related kinase (FRK), and Receptor tyrosine-protein kinase erbB-4 (HER4). Whereas the most-hypoactive STKs in adiposity include AMPKα1, MSK1, p70S6Kβ, Serine/threonine-protein kinase pim-1 (PIM1), PIM2, PKAα.

**Figure 6:**
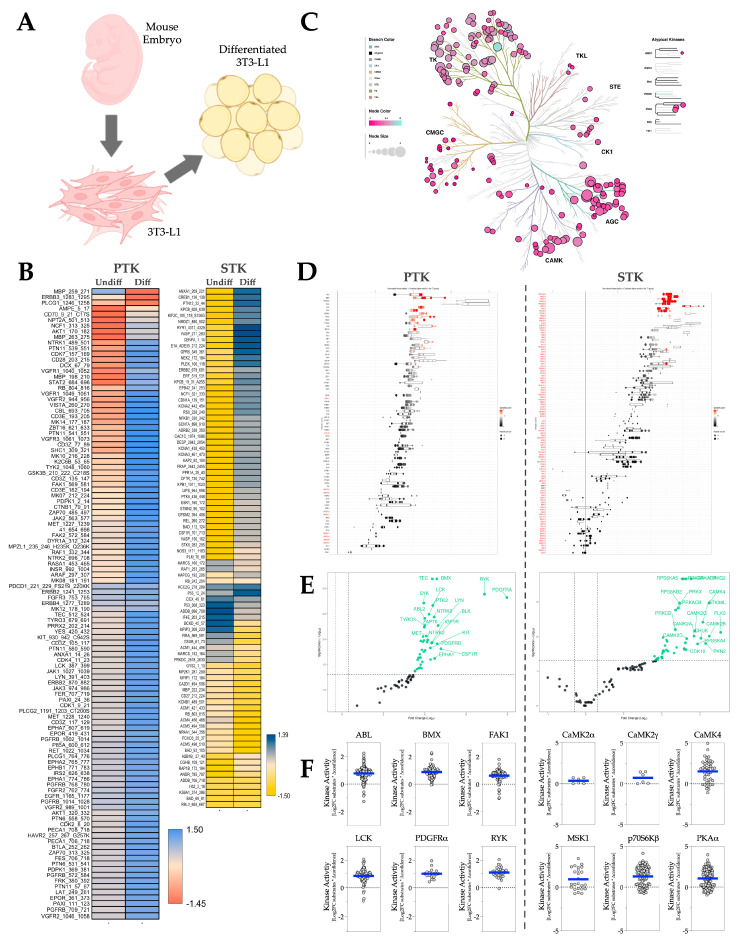
Murine adipocytes kinase activity profile in response to differentiation-induced adiposity. (**A**) Diagram illustrating the origin of 3T3-L1 cells and their morphological changes following differentiation. (**B**) Heatmaps showing average differentially phosphorylated PTK (**left**) and STK (**right**) substrates in undifferentiated (Undiff) or differentiated (Diff) 3T3L1 cells (N = 3 biological replicates). (**C**) Paralogous phylogenetic correlations between differentially activated kinases in response to differentiation-induced adiposity. The PTK and STK data from undifferentiated 3T3L1 or differentiated 3T3L1 were compared and analyzed. Node color represents the median kinase statistic (kinase activity), and node size represents the median final score (significance) on the bubble plot of the paralogous phylogenetic trees. (**D**) The individual PTK (**left**) and STK (**right**) activities, plotted using normalized kinase statistics, show the trend in kinase activity changes with adiposity. (**E**) Volcano plots showing the Log2 fold changes and significance of individual PTK (**left**) or STK (**right**). Green-colored kinases exhibit hyperactivity that passes both the significance and Log2FC cutoffs. (**F**) MEOW plots showing representative top-changed PTK (**left**) or STK (**right**) activities. The blue line in MEOW plots indicates the overall activity among all the included substrates.

3T3-L1, a mouse embryonic fibroblast cell line, is widely used for studying adipocytes (Figure 6A). To test if the kinome responsiveness in 3T3-L1 represents any of those in mouse adipose depots during adiposity progression, we differentiated 3T3-L1 cells and performed PamGene kinome analysis on them. Differentiation-induced adiposity increases phosphorylation of the majority of PTK and STK substrates (Figure 6B). The phyla tree shows that the overall activities of both PTK and STK increase with adiposity (Figure 6C). The waterfall and volcano plots show that activity for nearly all kinases increased (Figure 6D,E). Subsequently, we generated MEOW plots (Figure 6F) and identified that the most-changed PTKs in adiposity include ABL, BMX (Bone marrow tyrosine kinase gene in chromosome X protein), Focal adhesion kinase 1 (FAK1), LCK, Platelet-derived growth factor receptor alpha (PDGFRα), and Receptor-like tyrosine kinase (RYK). Whereas the most-changed STKs in adiposity include CaMK2α, calcium/calmodulin-dependent protein kinase gamma (CaMK2γ), CaMK4, MSK1, p70S6Kβ, and PKAα.

**Figure 7:**
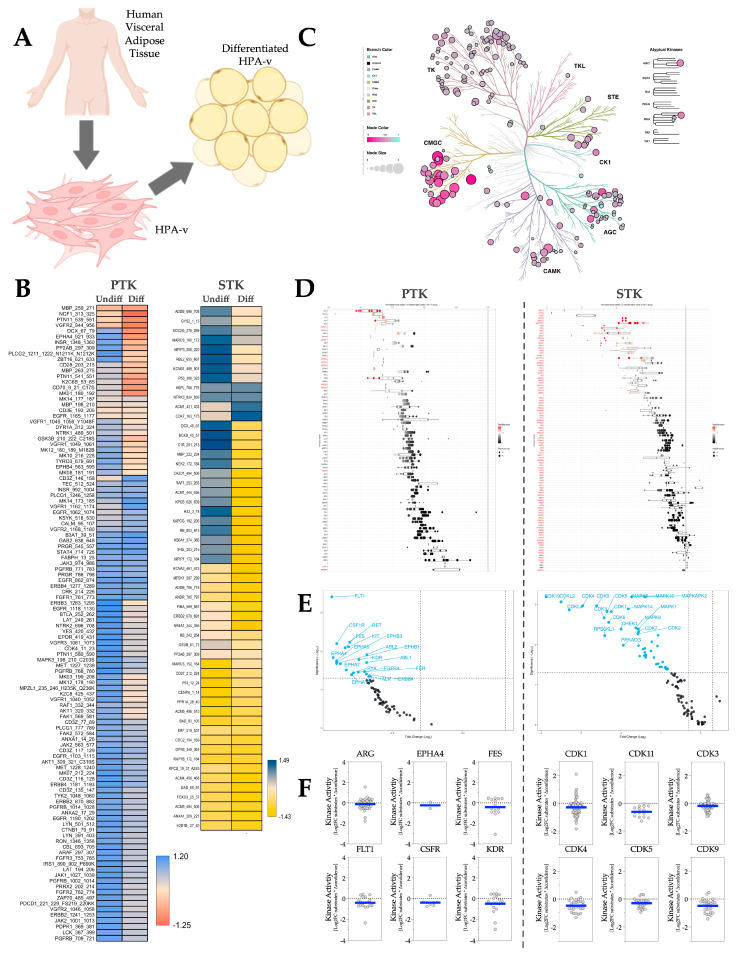
Human primary visceral adipocyte kinase activity profiles in response to differentiation-induced adiposity. (**A**) Diagram illustrating the origin of HPA-v cells and their morphological changes following differentiation. (**B**) Heatmaps showing average differentially phosphorylated PTK (left) and STK (right) substrates in undifferentiated (Undiff) or differentiated (Diff) HPA-v cells (N = 3 biological replicates). (**C**) Paralogous phylogenetic correlations between differentially activated kinases in response to differentiation-induced adiposity. The PTK and STK data from undifferentiated or differentiated HPA-v cells were compared and analyzed. Node color represents the median kinase statistic (kinase activity), and node size represents the median final score (significance) on the bubble plot of the paralogous phylogenetic trees. (**D**) The individual PTK (left) and STK (right) activities, plotted using normalized kinase statistics, show the trend in kinase activity changes with adiposity. (**E**) Volcano plots showing the Log2 fold changes and significance of individual PTK (left) or STK (right). Blue-colored kinases exhibit hypoactivity that passes both the significance and Log2FC cutoffs. (**F**) MEOW plots showing representative top-changed PTK (left) or STK (right) activities. The blue line in MEOW plots indicates the overall activity among all the included substrates.

HPA-v is a human primary visceral preadipocyte for studying human adipocytes (Figure 7A). To test whether the kinome responsiveness in human visceral adipocytes reflects VAT in mouse adipose depots during adiposity progression, we differentiated HPA-v cells and performed PamGene kinome analysis on them. Differentiation-induced adiposity resulted in decreased phosphorylation on most of the PTK and STK substrates (Figure 7B). The phyla tree shows that overall activity in both PTK and STK decreases as adiposity increases (Figure 7C). The waterfall and volcano plots show the same trend as the phyla tree (Figure 7D,E). The MEOW plots we generated (Figure 7F) exhibit that the most-changed PTKs in adiposity include Abelson murine leukemia viral oncogene homolog 2 (ARG), Ephrin type-A receptor 4 (EPHA4), Feline sarcoma/Fujinami avian sarcoma oncogene homolog (FES), Fms-like tyrosine kinase 1 (FLT1), CSFR, and Kinase insert domain receptor (KDR). Whereas the most-changed STKs in adiposity are from the cyclin-dependent kinase (CDK) family, which includes CDK1, CDK3, CDK4, CDK5, CDK9, and CDK11.

## 4. Discussion

Our kinome analysis shows that SCAT, represented by inguinal adipose tissue (iWAT), exhibits decreased PTK activity and increased STK activity. Adiposity increased the activities of both PTK and STK in mouse visceral adipose tissues, VAT, including eWAT and rWAT depots. In contrast, mWAT, also classified as VAT, exhibited a markedly distinct kinase activity profile compared to other VAT, in which the kinase activity was decreased. Brown adipose tissue (BAT) exhibited a unique kinase signature compared with both VAT and SCAT, with diverse changes in PTK activity and a decrease in STK activity. These findings show how depot-specific kinase activity profiles can differ from one another in adiposity.

While many kinases exhibit the same trend in activity with adiposity, we have shown that some show opposite activity across depots. BLK and ABL are two examples. In iWAT and mWAT, BLK is among the most hypoactive PTKs. In contrast, BLK is one of the most hyperactive PTKs in eWAT. Similarly, ABL exhibits the opposite activity in iWAT and eWAT. These kinases exemplify the diversity of signaling not only across adipose types, VAT and SCAT, but also within VAT depots. Given that PTKs are more likely to be upstream receptor kinases, their distinct activities in different depots may lead to entirely opposite signal cascades and physiological effects during adiposity. The kinome profile we generated is a rich resource for future signal-networking analysis and therapeutic target identification.

Among the adipose depots we analyzed, the changes in kinase profile in mWAT during adiposity were particularly intriguing. mWAT has been reported to be a unique adipose depot due to its immune-related interactions with the gut immune system, including greater immune cell infiltration under inflammatory conditions [24]. Local mWAT inflammation can also promote inflammation in fatty liver disease through lymphocyte activities [25]. Our findings show that BLK, EPHA1, FER, CSFR, FRK, and HER4 are the most hypoactivated PTKs in mWAT in response to adiposity. In addition, immune-regulating PTKs account for the majority of the most changed PTKs in mWAT, consistent with the highly immune-related characteristics of this depot. Notably, reduced BLK expression has been shown to increase proinflammatory cytokine levels and lymphocyte infiltration in mice [26], indicating that BLK hypoactivity may be a potential treatment target for mWAT-induced inflammation in adiposity. In addition, decreased EPHA1 activity may indicate T cell dysfunction, as EPHA1 plays a key role in T cell function [27]. Since EPHA1 is the most-changed PTK specific to mWAT, it may serve as a signature mediator and a potential therapeutic target for mWAT-related immune disturbances during adiposity. Although more studies are needed to examine the practical and clinical use of the kinases identified in our study, the kinome profile has shown its potential for rapid therapeutic target identification and for facilitating therapeutic development.

Given that a significant portion of existing research on the adipose tissue response to increased adiposity is conducted in vitro and is not depot-specific, our kinome analysis using the well-established, widely used adipocyte cell lines 3T3-L1 and HPA-v helps link our new findings to prior research in this domain. The 3T3-L1 cell line exhibits kinase activity trends comparable to those observed in eWAT, with both PTK and STK activities increasing during adiposity. This suggests that data derived from 3T3-L1 models more accurately reflect eWAT activity than those from other adipose depots. Importantly, HPA-v demonstrates a kinase activity profile similar to that observed in mWAT during adiposity, indicating that prior findings from HPA-v models are more likely to represent mWAT responses during increased adiposity. These insights may be valuable in guiding the design of future in vitro studies.

As with all research endeavors, our study has certain limitations that we wish to acknowledge to guide future investigations towards a more comprehensive understanding of adiposity and kinase signaling mechanisms. A notable drawback is that the study is predominantly descriptive, lacking detailed analysis of variations in kinase activity or causal testing. Human validation was limited to a single cell model (HPA-v preadipocytes), with no data from in vivo human tissues. The article indicated that mouse adipocytes (3T3-L1) demonstrated increased kinase activity, whereas human adipocytes showed decreased activity. This apparent contradiction remains unelucidated and may have biological significance, thereby necessitating further research to clarify these disparities. Future studies elucidating the interactions between the preadipocyte microenvironment and associated pathways during metabolic dysfunction would be highly advantageous.

## 5. Conclusions

This study found variations in kinase activity across white and brown adipose tissues, mouse adipocytes, and human primary adipocytes in relation to adiposity development. Fat accumulation at different anatomical locations fulfills distinct physiological and metabolic roles. Moreover, it identifies kinases that may serve as biomarkers of adiposity within specific fat depots. These findings enhance our understanding of obesity-related pathways and support the identification of depot-specific therapeutic targets to mitigate adiposity. Building on prior in vitro investigations of adiposity, this research provides a broader context and highlights potential targets for future anti-obesity interventions.

## Figures and Tables

**Table 1:** Animal and tissue weights.

	**NCD (Lean)**	**HFD (Obese)**	
	Mean ± SD	Mean ± SD	*p*-value
Body Weight (g)	25.45 ± 1.66	46.80 ± 3.41	<0.0001
Fat Mass (g)	1.00 ± 0.31	14.34 ± 2.88	<0.0001
Lean Mass (g)	23.43 ± 2.05	30.48 ± 1.49	<0.0001
Blood Glucose (mg/dL)	123.50 ± 16.85	159.00 ± 13.53	0.0021
iWAT (g)	0.15 ± 0.03	1.61 ± 0.52	0.0003
eWAT (g)	0.25 ± 0.06	1.22 ± 0.37	0.0004
mWAT (g)	0.14 ± 0.07	0.71 ± 0.27	0.0012
rWAT (g)	0.07 ± 0.01	1.11 ± 0.27	<0.0001
BAT (g)	0.08 ± 0.01	0.19 ± 0.02	<0.0001
Liver (g)	0.95 ± 0.08	1.63 ± 0.30	0.0006
YffHeart (g)	0.13 ± 0.02	0.19 ± 0.03	0.0005
Pancreas (g)	0.12 ± 0.05	0.40 ± 0.18	0.0055
Spleen (g)	0.06 ± 0.01	0.11 ± 0.04	0.0061
Kidney (g)	0.31 ± 0.03	0.45 ± 0.04	<0.0001
Body weight normalization	**NCD (Lean)**	**HFD (Obese)**	
	Mean ± SD	Mean ± SD	*p*-value
Fat Mass/BW	0.0399 ± 0.0127	0.3042 ± 0.0375	<0.0001
Lean Mass/BW	0.9194 ± 0.0262	0.6539 ± 0.0516	<0.0001
iWAT/BW	0.0058 ± 0.0012	0.0339 ± 0.0088	<0.0001
eWAT/BW	0.0096 ± 0.0019	0.0263 ± 0.0080	0.0004
mWAT/BW	0.0055 ± 0.0024	0.0149 ± 0.0050	0.0015
rWAT/BW	0.0027 ± 0.0005	0.0234 ± 0.0040	<0.0001
BAT/BW	0.0030 ± 0.0003	0.0041 ± 0.0005	0.0006
Liver/BW	0.0371 ± 0.0013	0.0348 ± 0.0051	0.3105
Heart/BW	0.0050 ± 0.0003	0.0040 ± 0.0006	0.0040
Pancreas/BW	0.0046 ± 0.0019	0.0084 ± 0.0034	0.0342
Spleen/BW	0.0022 ± 0.0003	0.0023 ± 0.0006	0.6813
Kidney/BW	0.0121 ± 0.0003	0.0096 ± 0.0008	<0.0001

## Data Availability

Complete kinome reports and raw data files are available on Figshare (URL: https://doi.org/10.6084/m9.figshare.32108836). This study did not generate any unique code.

## Abbreviations

The following abbreviations are used in this manuscript:

MASLD	Metabolic dysfunction-associated steatotic liver disease
iWAT	Inguinal white adipose tissue
eWAT	Epididymal white adipose tissue
BAT	Brown adipose tissue
rWAT	Retroperitoneal white adipose tissue
mWAT	Mesenteric white adipose tissue
VAT	Visceral adipose tissue
SCAT	Subcutaneous adipose tissue
NCD	Normal chow diet
HFD	High fat diet
FBS	Fetal Bovine Serum
M-PER	Mammalian Extraction Reagent
PTK	Protein Tyrosine Kinase
STK	Serine-Threonine Kinase
ATP	Adenosine triphosphate
CCD	Charge-coupled device
LED	Light-emitting diode
K.R.S.A	Kinome Random Sampling Analysis
CV	Coefficient of variation
FC	Fold change
UKA	Upstream Kinase Analysis
BW	Body weight
ABL	Abelson murine leukemia viral oncogene homolog 1
BLK	B lymphocyte kinase
FYN	Proto-oncogene Syn
HCK	Hematopoietic cell kinase
LCK	Leukocyte C-terminal Src kinase
LYN	Lck/Yes-related novel protein tyrosine kinase
CaMK2α	CaM kinase II subunit alpha
CaMK2δ	CaM kinase II subunit delta
IKKα	I-kappa-B kinase alpha
p70S6Kβ	S6K-beta-2
PKAα	cAMP-dependent protein kinase catalytic subunit alpha
PKCα	Protein kinase C alpha
FRK	FYN-related kinase
HER3	Receptor tyrosine-protein kinase erbB-3
HER4	Receptor tyrosine-protein kinase erbB-4
RON	Macrophage-stimulating protein receptor
AMPKα1	5′-AMP-activated protein kinase catalytic subunit alpha-1
PKG1	cGMP-dependent protein kinase 1
PKG2	cGMP-dependent protein kinase 2
PKN1	Serine/threonine-protein kinase N1
EPHA10	Ephrin type-A receptor 10
EPHA5	Ephrin type-A receptor 5
EPHA4	Ephrin type-A receptor 4
FGR	Proto-oncogene c-Fgr
JAK3	Janus kinase 3
MAP2K1	Dual specificity mitogen-activated protein kinase kinase 1
ROS	Receptor tyrosine kinase c-ros oncogene 1
ANPα	Atrial natriuretic peptide receptor type A
CDKL1	Cyclin-dependent kinase-like 1
CHK2	Checkpoint kinase 2
DYRK1A	Dual specificity YAK1-related kinase
mTOR	Mammalian target of rapamycin
p38β	Mitogen-activated protein kinase 11
CTK	CSK homologous kinase
MAP2K7	Dual specificity mitogen-activated protein kinase kinase 7
SRC	Proto-oncogene c-Src
YES	Proto-oncogene c-Yes
CaMK4	Calcium/calmodulin-dependent protein kinase type IV
MSK1	S6K-alpha-5
PIM1	Serine/threonine-protein kinase pim-1
PIM2	Serine/threonine-protein kinase pim-2
EPHA1	Ephrin type-A receptor 1
FER	Feline encephalitis virus-related kinase FER
CSFR	Macrophage colony-stimulating factor 1 receptor
BMX	Bone marrow tyrosine kinase gene in chromosome X protein
FAK1	Focal adhesion kinase 1
PDGFRα	Platelet-derived growth factor receptor alpha
RYK	Receptor like tyrosine kinase
CaMK2γ	Calcium/calmodulin-dependent protein kinase gamma
ARG	Abelson murine leukemia viral oncogene homolog 2
FES	Feline sarcoma/Fujinami avian sarcoma oncogene homolog
FLT1	Fms-like tyrosine kinase 1
KDR	Kinase insert domain receptor
CDK	Cyclin-dependent kinase
